# Extending the functional characteristics of naturally occurring autoantibodies against β-Amyloid, Prion Protein and α-Synuclein

**DOI:** 10.1371/journal.pone.0202954

**Published:** 2018-08-29

**Authors:** Alexandra Albus, Maike Gold, Jan-Philipp Bach, Monika Burg-Roderfeld, Marit Jördens, Yvonne Kirchhein, Yannick Kronimus, David Mengel, Inga Zerr, Richard Dodel

**Affiliations:** 1 Chair of Geriatrics, University Hospital Essen, University Duisburg-Essen, Essen, Germany; 2 Department of Neurology, Philipps-University, Marburg, Germany; 3 Department of Neurology, RWTH Aachen, Aachen, Germany; 4 Department of Hematology and Immunology, Justus-Liebig-University, Giessen, Germany; 5 Department of Neurology, University Goettingen, Goettingen, Germany; IRCCS - Mario Negri Institute for Pharmacological Research, ITALY

## Abstract

**Background:**

Abnormal aggregation of proteins induces neuronal cell loss in neurodegenerative disorders such as Alzheimer’s Disease, Creutzfeldt-Jakob Disease and Parkinson’s Disease. Specific stimuli initialize conformational changes in physiological proteins, causing intra- or extracellular protein aggregation. We and other groups have identified naturally occurring autoantibodies (nAbs) as part of the human antibody pool that are able to prevent peptide fibrillation. These nAbs show a rescue effect following exposure of toxic aggregates on neurons, and they support microglial uptake of aggregated peptides.

**Objective:**

Identification of a putative common epitope among the relevant proteins β-Amyloid, α-Synuclein and Prion Protein for the respective nAbs.

**Material and methods:**

Binding affinity between the aforementioned proteins and nAbs was tested by Dot Blot, ELISA and SPR-technology. Furthermore, the functionality of the protein-nAbs-complexes was studied in Thioflavin-T assays and microglial uptake experiments to study dependent inhibition of protein aggregation and enhancement of Fcγ mediated uptake by microglial cells.

**Results:**

β-Amyloid and Prion Protein fragment showed considerable binding affinity and functional efficacy for all applied nAbs. Thereby, no significant difference within the different nAbs was detected. In contrast, α-Synuclein was bound exclusively by nAbs-α-Synuclein, which was reproduced in all binding studies. Surprisingly, functional assays with α-Synuclein revealed no significant effect of nAbs in comparison to IVIg treatment. However, all applied nAbs as well as IVIg show a minimal functionality on the microglial uptake of α-Synuclein.

**Conclusion:**

nAbs-Aβ, nAbs-PrP possibly display comparable affinity to the same structural epitope within Aβ and PrP106-126 A117V whereas the epitope recognized by nAbs-α-Syn is only present in α-Syn. The structural similarity of Aβ and PrP fragment promotes the outline for an efficient antibody for the treatment of several neurodegenerative disorders and extend the functional characteristics of the investigated nAbs.

## Introduction

The pathology of neurodegenerative disorders including Alzheimer’s Disease (AD), Creutzfeldt-Jakob Disease (CJD) and Parkinson’s Disease (PD) is characterized by a deposition of aggregated proteins in distinct areas within the brain, which causes destabilization of neuroplasticity followed by neuronal cell loss, resulting in specific clinical impairments [1]. Each of the diseases is defined by the misfolding of a characteristic peptide/protein.

The aggregation and subsequent extracellular deposition of β-Amyloid (Aβ) is the hallmark in AD [2]. Aβ originates from the larger Amyloid Precursor Protein (APP) by cleavage through β- and γ-secretases, resulting in primarily Aβ_1–40_ or Aβ_1–42_. Aβ has the propensity to form oligomers and fibrils, which has been shown to induce neurotoxicity [3,4].

In CJD, cellular Prion protein (PrP^c^) changes its conformation by a still unknown mechanism into pathologic Prion Scrapie Protein (PrP^sc^). PrP^sc^ adopts a predominant β-pleated conformation that results in a water insoluble protein and resistance to protease digestion [5]. CJD patients show extracellular PrP^sc^ aggregates around neurons in affected brain areas. These amyloid-like plaques are thought to induce neuronal dysfunction [6,7].

α-Synuclein (α-Syn) plays a crucial role in PD by its intracellular deposition in the substantia nigra pars compacta and the predominant protein in intracellular Lewy body inclusions [8]. Misfolding of α-Syn can be found in patients with mutations within the SNCA gene, the α-Syn coding gene, or can result from posttranscriptional modification of α-Syn. Formation of α-Syn into prefibrillar oligomers, so called protofibrils, may induce neurotoxicity [9,10].

In this context, we and other groups have identified naturally occurring autoantibodies (nAbs) as part of the human antibody pool [11–14]. nAbs were first mentioned in 1959 and are often described as preimmune antibodies, which are produced in the absence of an antigen stimulus [15,16]. These nAbs are secreted by B1-cells [17], which are generated early in the development from fetal liver precursor cells and proliferate independently of T-cells [18]. Thus, nAbs are present at a constant level throughout the lifespan. Previous studies demonstrated that individuals suffering from a neurodegenerative disease such as AD, CJD or PD show an altered level of nAbs in their serum, which indicates a beneficial effect for these nAbs [11,13,19–21].

Recent preclinical data indicate that nAbs are able to prevent PrP fibrillation [22] and enhance the microglial uptake of aggregated Aβ and PrP *in vitro* [23–26]. Interestingly, nAbs-Aβ showed a binding to oligomerized Aβ but not to monomers or fibrils [27,28]. In addition, nAbs-Aβ exert positive effects in transgenic mouse models of AD. Treatment with nAbs-Aβ induces a reduction of plaques in young TgCRND8 mice [27] and reduces cytokine secretion in Tg2576 mice [29]. Tests in APP/PS1 transgenic mice confirmed these findings [26]. Furthermore, nAbs-Aβ-treated Tg2576 mice exhibit an improvement of their cognition in behavioral tests [29].

Until now, no data are available that address the differential specificity of those nAbs. To investigate the concordant effects of specific nAbs *in vitro*, we tested binding affinity and function among different nAbs in order to identify their distinctive epitope specificity. In addition, we hypothesize about a possible structural similarity among the epitopes targeting the different aggregated proteins.

## Materials and methods

### Peptides

Aβ1–42 and Aβ1-42-FITC (Bachem, Bubendorf, Switzerland), PrP106-126 A117V and PrP106-126 A117V-FITC (further stated as PrP fragment, PSL GmbH, Heidelberg, Germany) and α-Syn (rPeptide, Bogart, USA) were used. All Aβ experiments (except for ELISA and ThT assays) were carried out with oligomerized Aβ1–42 by applying an established protocol from our lab [29]. For ELISA and ThT assays monomeric Aβ was used. For PrP experiments, a short fragment of the prion protein with a single mutation (PrP106-126 A117V), was applied, which was successfully used previously in aggregation studies and already showed oligomerization [30,13,24]. Furthermore, this PrP version exhibits some pathological characteristics of PrP^sc^, like fibril formation [31]. The used PrP is further stated as aggregated PrP fragment in case we used oligomerized peptide. For ThT assay monomeric PrP fragment was used. α-Syn experiments were carried out with monomeric peptide using freshly dissolved full-length protein as already published from our workgroup [30]. For microglial uptake assays peptides had to be FITC labeled. Aβ1–42—and PrP-FITC fragment peptides were purchased by the manufacturer (see above). Only α-Syn had to be FITC labeled by means of a labeling kit (Lightning-Link®-Fluorescein, Innova Bioscience, Cambridge, United Kingdom). For this, the α-Syn had to be exempted from the Trifluoroacetic acid-salt, which it contains after the production process, by dialysis in water. Afterwards, the solution was freeze-dried and was brought to a concentration of 20 mg/mL with PBS. The obtained solution was labeled following the manufacturer´s protocol.

### Purification of nAbs

nAbs were isolated from intravenous immunoglobulins (IVIg) via affinity chromatography as outlined previously [27]. For this, we used commercially available IVIg (Octagam 10%, Octapharma, Langenfeld, Germany). IVIg preparations consist of pooled antibodies of several blood donations and thereby reflect the antibody pool of healthy persons. Briefly, disposable chromatography columns were packed with iodoacetyl gel (Thermo Scientific, Rockford, IL, USA) coupled with peptide fragments of the Aβ-, PrP- or α-Syn-protein. IVIg (1:1 in PBS) was loaded on the columns overnight at 4 °C. The unbound fraction (IVIg depleted of nAbs-Aβ, -PrP or -α-Syn) passed through the columns. After loading IVIg several times, the bound IgG fraction was released and collected by passing elution buffer (50 mM glycine with pH 2.8) through the column. The pH of the nAbs was further normalized with TRIS to 7.4. nAbs were washed with PBS and concentrated by centrifugation using an ultrafiltration filter (Vivaspin® 20, Sartorius AG, Göttingen, Germany) to 3 mg/ml. In all experiments IVIg was used as reference control to subtract a possible general effect of the IgG mixture.

### Cell culture

Primary microglial cells were cultured as described previously [24]. Briefly, mesencephalons of embryonic mice from embryonic day 13.5 were used. The mesencephalons were collected in 2 ml of Leibovitz L-15 medium (PAA Laboratories, Pasching, Austria) and homogenized by gently pipetting up and down several times. L-15 medium (5 ml) was added, the cell solution was left for 10 min to remove debris and 5 ml of the supernatant was transferred into a new tube. The cell dispersion was centrifuged for 5 min at 300xg and the supernatant was discarded. The remaining pellet was resuspended in 1 ml Dulbecco’s modified Eagle’s medium (DMEM with L-Glutamine; Lonza, Basel, Switzerland) supplemented with 10% fetal calf serum (FCS; PAA Laboratories) and 100 U/ml penicillin and 100 μg/ml streptomycin (Lonza). The cells were cultured in polyethylenimine (PEI-) coated 6-well plates. To improve the yield of microglial cells, 10 ng/ml GM-CSF (Roche, Basel, Switzerland) was added to the medium. Cells were replated without a preceding trypsinization step and cultured 14 days until experimental use. Cells were replated onto PEI-coated 24-well plates at a density of 1–2 x 10^5^ cells per ml. Fully 90–95% of the cells were microglial cells according to staining with CD11b antibody. All animal procedures were approved by the office of the district president and the Institutional Animal Care and Use Committee of the University of Marburg.

### BV-2 culture

The murine microglial cell line BV-2 was kindly provided by Jens Neumann, Magdeburg, Germany and cultured as previously described [31]. Cells were cultured in a T-25 flask in 5 ml DMEM supplemented with 10% FCS and 100 U/ml penicillin and 100 μg/ml streptomycin. For experiments, cells were plated in a 24-well plate with a density of 2–3 x 10^5^ cells per ml.

### Microglial uptake

To analyze microglial uptake FACS analysis was done to determine FITC-fluorescence intensity of FITC-conjugated peptides. Microglial uptake of aggregated PrP fragment was carried out as described previously [24]. PrP-FITC fragment at 150 μM was incubated in PBS for 48 h at 37 °C to generate aggregates, which were then incubated at a concentration of 10 μM with 0.16 μM nAbs/IVIg for 1 h in serum-free DMEM before treatment of primary microglial cells for 3 h. The cells were washed 3 times with ice-cold PBS, harvested and transferred to FACS tubes (Sarstedt AG & Co., Nuembrecht, Germany). For FACS analysis, cells were washed with FACS buffer (PBS with 0.1% FCS) and probed with 1:1000 APC-conjugated CD11b antibody (eBioscience Inc., San Diego, USA) for 20 min at 4 °C protected from light. The cells were washed with FACS buffer for a second time and stained before analysis with HOECHST 33258 (Sigma, St Louis, MO, USA). Microglial uptake was measured with a LSR II flow cytometer (Becton, Dickinson and Company, Franklin Lakes, USA). For analysis, the software Flow Jo (Tree Star Inc., Ashland, USA) was used. Only CD11b positive and HOECHST 33258 negative cells were used for microglial uptake.

The uptake of α-Syn was measured also by FACS analysis of FITC labeled protein. For this, primary microglial cells were pretreated with 0.1 μM nAbs/ IVIg in serum-free DMEM for 1 h and further incubated with FITC-conjugated α-Syn at 0.5 μM for 2 h. The following treatment was identical to PrP uptake.

For Aβ uptake, BV-2 cells were used. Oligomerized FITC-conjugated Aβ_1–42_ at 1 μM was preincubated with 0.1 μM nAbs/ IVIg in serum-free DMEM for 1 h and further incubated with BV-2 cells for 3 h. The cells were washed with PBS. The treatment was identical to that described for PrP uptake, except for the CD11b staining, which is not necessary in a monoclonal microglial cell line.

### Dot blot

Peptides were dotted in a decreasing concentration pattern (0.33, 0.165, 0.083, 0.041, 0.021 mg/ml) on a nitrocellulose membrane (0.45 μm, Merck KGaA, Darmstadt, Germany), blocked with Roti-Block reagent (Roth, Karlsruhe, Germany) for 1 h at RT, followed by an overnight incubation with 1 μg/ml nAbs/IVIg solution in Roti at 4 °C. The membrane was washed in TBST (Tris-buffered saline with 0.05% Tween 20) and incubated with 1:500,000 HRP-conjugated anti-human detection antibody (Thermo Fisher Scientific Inc., Rockford, USA). Control stainings were performed by using the secondary antibody alone. Visualization was performed by using a HRP visualization kit (Super Signal West Dura Extended Duration Substrate, Thermo Fisher Scientific Inc., Rockford, USA) followed by exposure to X-ray film.

### ELISA

The surface of a 96-well plate (Immulon® 2 HB, U bottom, high bind, Thermo Fisher Scientific Inc. Rockford, USA) was coated overnight with 20 μg/ml (10 μM) PrP fragment or 8 μg/ml (0.57 μM) α-Syn dissolved in PBS. Unspecific binding was prevented by incubation with blocking buffer (Superblock®, Thermo Fisher Scientific Inc., Rockford, USA with 1% Tween 20) for 24 h. The plates were washed three times with washing solution (PBS with 0.01% Tween 20) and then incubated with decreasing concentrations of nAbs/IVIg for 1 h at RT. As secondary antibody, a goat-anti-human antibody (Thermo Fisher Scientific Inc., Rockford, USA) was diluted 1:5,000 in blocking buffer and incubated for 1 h on a shaker. After washing TMB-solution (Thermo Fisher Scientific, Rockford, USA) was applied for 15 min for visualization, and the reaction was stopped with sulfuric acid (5% H_2_SO_4_, Sigma-Aldrich® Corporation, St. Louis, USA) as stop solution. Signals were measured at 450 nm with a plate reader (Tecan Infinite M200), and the background signal was subtracted.

For Aβ ELISA, the surface of a high-binding 96-well plate (Immunolon, Thermo Scientific, Rockfort, IL; USA) was coated with 10 μg/ml (2.35 μM) Aβ_1–42_ dissolved in 0.2 M sodium phosphate buffer (pH 7.4) overnight at 4 °C. For control purposes, half of the plate was incubated with 0.2 M sodium phosphate buffer only to determine the unspecific background signal. The plates were washed three times with washing solution (PBS with 0.01% Tween 20). Blocking was performed using Roti-Block reagent supplemented with 0.1% Tween-20 (Applichem GmbH, Darmstadt, Germany) overnight at 4 °C. The plates were washed 3 times and then incubated with decreasing concentrations of nAbs/IVIg for 1 h at RT. After washing, secondary antibody (goat anti-human IgG, biotinylated, Dianova, Hamburg, Germany) was diluted 1:20,000 in blocking buffer and incubated for 1 h. Following washing and a second blocking step for 1 h at RT, streptavidin-peroxidase (R&D Systems, Minneapolis, MN, USA) was used at 1:200 in blocking reagent and added to the plates and kept in the dark at RT for 20 min. After a final washing step, TMB-solution (Merck, Darmstadt, Germany) was applied for 20 min, and the reaction was stopped with sulfuric acid (5% H_2_SO_4_) as stop solution. Signals were measured at 450 nm with a plate reader (Tecan Infinite M200, Crailsheim, Germany), and the background signal was subtracted.

### SPR spectroscopy

SPR analysis was performed on a protein interaction array system (ProteOn XPR36, Bio-Rad, Munich, Germany) at 25 °C. Different peptides (25 μg/ml solutions in 10 mM acetate buffer pH 4.5) were immobilized covalently onto parallel channels of a GLH sensor chip via amine coupling according to the suppliers’ protocol. Remaining active sites on the sensor chip were blocked by treatment with 1 M ethanolamine HCl pH 8.5. Immobilization and deactivation steps were carried out at a flow rate of 30 μl/min and 5 min contact time. PBS containing 0.005% Tween 20 was used as running buffer. For interaction analysis, five concentrations of nAbs (0.125, 0.25, 0.5, 1.0 and 2.0 μM in running buffer) were injected in parallel channels perpendicularly to the peptide channels at a flow rate of 25 μl/min and 6 min contact time followed by 5 min dissociation. After every interaction, the chip surface was regenerated by injection of 10 mM glycine buffer pH 2.5 at a flow rate of 100 μl/min and 18 s contact time. The interaction curves were double referenced using the ProteOn Manager software (BioRad, Munich, Germany) by subtracting interspot and buffer injection data. The kinetic constants of the interactions were calculated by the Langmuir kinetic model function of the software.

### ThT assay

Fibril formation assays were carried out as described previously [24]. Briefly, Aβ_1–42_ at 50 μM was incubated with or without 2.5 μM nAbs/ IVIg. For PrP fragment a working concentration of 150 μM was used. The fibrillation status was measured after a 24 h incubation in a black 96-well plate (Greiner Bio-One GmbH, Frickenhausen, Germany) by adding 80 μl glycine buffer (50 mM, pH 9.2) and 10 μl ThT (4 mM) to 10 μl of the fibrillation mixture.

### Statistical analysis

For statistical analysis we used ANOVA with the Bonferroni post-hoc test (p < 0.05 (*)). If not stated otherwise, the mean ± standard deviation of the mean are shown.

## Results

### Differential binding and function of β-Amyloid following exposure to nAbs-Aβ, nAbs-PrP and nAbs-α-Syn

**Fig 1:**
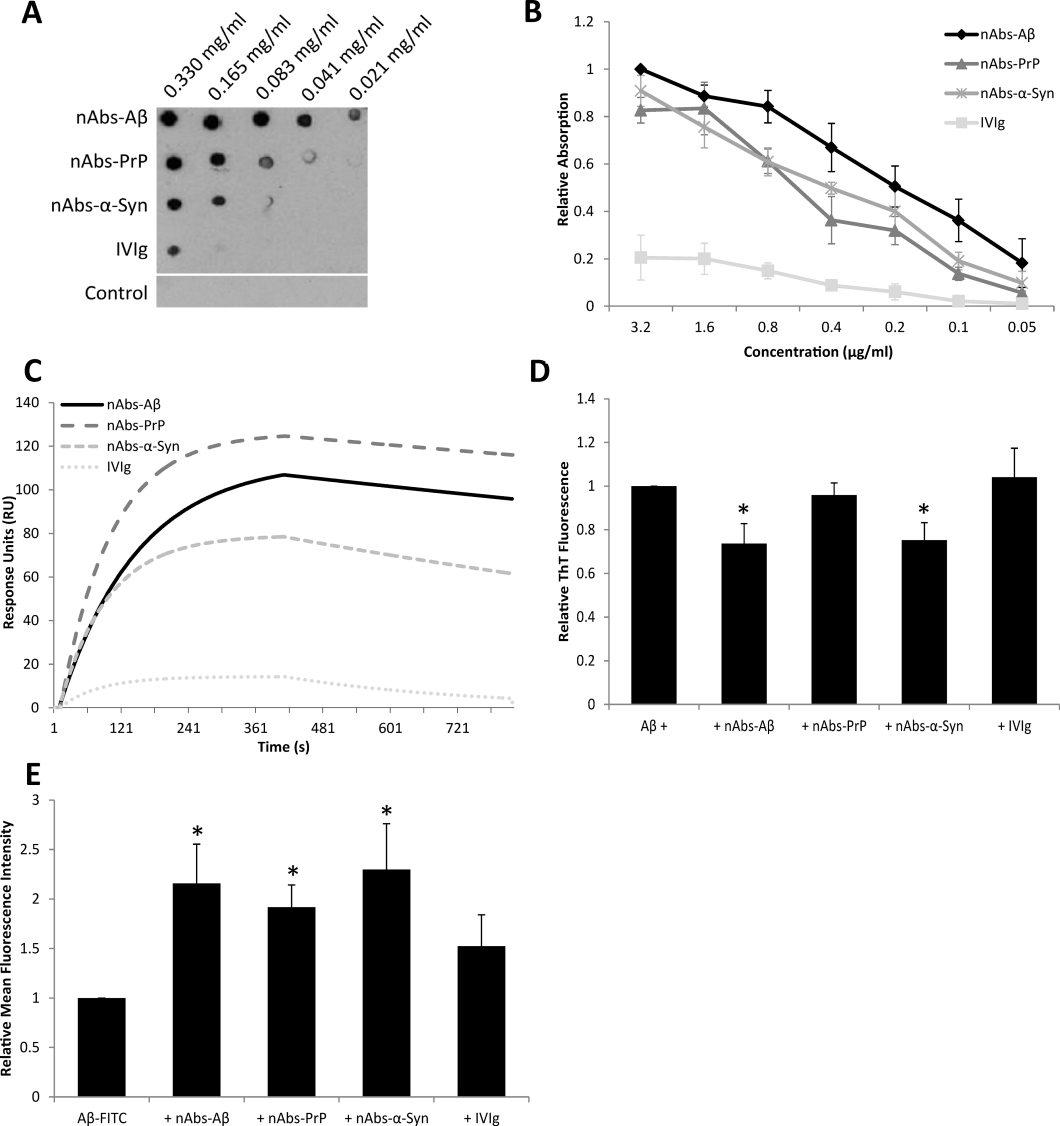
Binding and function of nAbs on Aβ_1–42_. (A) Interaction between nAbs and Aβ_1–42_ in Dot Blot. Aβ_1–42_ oligomers were dotted in a decreasing concentration pattern, and the membrane was further incubated in nAbs-solution. To exclude unspecific binding, the membrane was also incubated with secondary antibody only (control; anti-human 1:500,000). One out of three independent experiments is shown. (B) Measurement of Aβ_1–42_ binding to nAbs by ELISA. The surface of a 96-well plate was coated with Aβ_1–42_ and incubated with different concentrations of nAbs/ IVIg. The data were normalized to the highest nAbs-Aβ concentration. (C) SPR sensorgram of Aβ_1–42_ interaction. Aβ_1–42_ was used as ligand whereas nAbs were used as analytes. The graph shows the different binding affinities between nAbs/ IVIg and the Aβ_1–42_ peptide. Interaction was measured between about 1000 RUs Aβ_1–42_ and 0.5 μM nAbs/ IVIg. Interactions are presented after a Langmuir fitting. (D) ThT-assay of Aβ_1–42_ following treatment with different nAbs for quantification of fibril formation. Aβ was incubated with or without nAbs/ IVIg. (E) FACS analysis of Aβ_1–42_ uptake by microglial BV-2 cells in the presence of different nAbs. Aβ_1–42_ was oligomerized first and then preincubated with nAbs/ IVIg before treatment of BV-2 cells. For B, D, E mean values ± SD of three independent experiments are shown. For every assay, IVIG was used as a reference control.

All applied nAbs showed a binding to Aβ_1–42_ in Dot Blot (Fig 1A) as well as in ELISA (Fig 1B) and SPR (Fig 1C) experiments. In Dot Blot experiments, however, nAbs-Aβ showed the highest avidity to Aβ_1–42_ with considerably lower binding avidity of nAbs-PrP and nAbs-α-Syn. The control staining of the secondary antibody alone showed no unspecific binding to the peptide. Only weak interactions were detected using the same concentrations of IVIg, which were set as a reference control. These findings were confirmed by ELISA (Fig 1B). Aβ was detected by all nAbs/IVIg with different avidities even at low concentrations of nAbs/IVIg (0.8 μg/ml). Furthermore, nAbs were significantly higher in their avidity in comparison to the reference control IVIg, resulting in a higher binding signal (nAbs-Aβ: *p* = 0.0001; nAbs-PrP: *p* = 0.0006; nAbs-α-Syn: *p* = 0.0004).

The SPR test enables the real-time interaction of Aβ and nAbs. Surprisingly, nAbs-PrP exhibited slightly higher RU and KD values than nAbs-Aβ (Fig 1C). However, all nAbs interacted strongly with Aβ_1–42_ and were considerably more specific than IVIg, as shown in the kinetic parameters (Table 1A). The association and dissociation of the different nAbs to/from Aβ were nearly concordant to each other and reflect a similar binding affinity. The noise during association and dissociation measurement was quite low, which supports a precise interaction.

**Table 1:** Kinetic parameters of SPR measurements.

	ka [Ms^-1^]	kd [s^-1^]	KD [M]	Rmax [RU]	Rmax Error [RU]
***A* Aβ**					
nAbs-Aβ	1.54·10^4^	2.97·10^−4^	1.93·10^−8^	117.57	4.39·10^−1^
nAbs-PrP	2.41·10^4^	1.97·10^−4^	8.19·10^−9^	128.24	4.24·10^−1^
nAbs-α-Syn	2.46·10^4^	6.61·10^−4^	2.69·10^−8^	83.49	3.78·10^−1^
IVIg	2.48·10^4^	3.27·10^−3^	1.32·10^−7^	18.11	3.61·10^−1^
***B* α-Syn**					
nAbs-Aβ	1.50·10^−7^	6.40·10^−7^	4.28	5.84·10^−6^	1.59·10^17^
nAbs-PrP	2.12·10^−7^	8.98·10^−7^	4.23	2.58·10^−5^	4.58·10^9^
nAbs-α-Syn	6.86·10^3^	1.33·10^−3^	1.94·10^−7^	240.36	3.36
IVIg	9.92·10^7^	2.37·10^1^	2.39·10^−7^	1.17	7.90·10^−2^

(A) Binding affinity with Aβ_1–42_. An SPR chip was coated with 25 μg/ml oligomerized Aβ_1–42_. Thereby, about 1000 RUs were bound covalently on the surface. nAbs/ IVIg were used in a decreasing concentration pattern, starting with 2 μM for 360 sec on the chip, followed by dissociation. KD values show the strength of the antibody-antigen interaction. A KD value between 10^−5^ and 10^−8^ M represents a strong interaction. Interaction of 0.5 μM nAbs/ IVIg and Aβ is shown. (B) Binding affinity with α-Syn. During immobilization step with 25 μg/ml α-Syn about 2000 RUs were covalently bound on the chip surface. Decreasing concentrations of nAbs/ IVIg were added to the α-Syn coated SPR chip for 360 sec, followed by dissociation. KD values indicate the strength of antibody-antigen binding. The table shows the interaction of 0.5 μM nAbs/ IVIg with α-Syn. *ka* association constant, *kd* dissociation constant, *KD* equation binding constant, *Rmax* maximal response units, *Rmax error* standard error of Rmax.

Using functional assays, fibrillation of Aβ using ThT was significantly inhibited by nAbs-Aβ with a power of 26% (*p* = 0.03) and nAbs-α-Syn with a power of 24% (*p* = 0.03) in comparison to IVIg (Fig 1D). Therefore, nAbs-/IVIg-samples were normalized to the untreated sample as reference control. In contrast, fibrillation of Aβ was not significantly prevented by nAbs-PrP treatment (*p* = 0.38).

Microglial uptake of Aβ was tested using BV-2 cell cultures: Peptide uptake was increased by nAbs-Aβ (*p* = 0.05), nAbs-PrP (*p* = 0.04) and nAbs-α-Syn (*p* = 0.05)(Fig 1E), whereas IVIg treatment as the reference control revealed only slight effects. Compared to untreated Aβ-FITC, all nAbs could enhance the uptake with a factor between 1.9 (nAbs-PrP) and 2.2 (nAbs-Aβ and nAbs-α-Syn).

### Differential binding and function of prion peptide fragment following exposure to nAbs-Aβ, nAbs-PrP and nAbs-α-Syn

**Fig 2:**
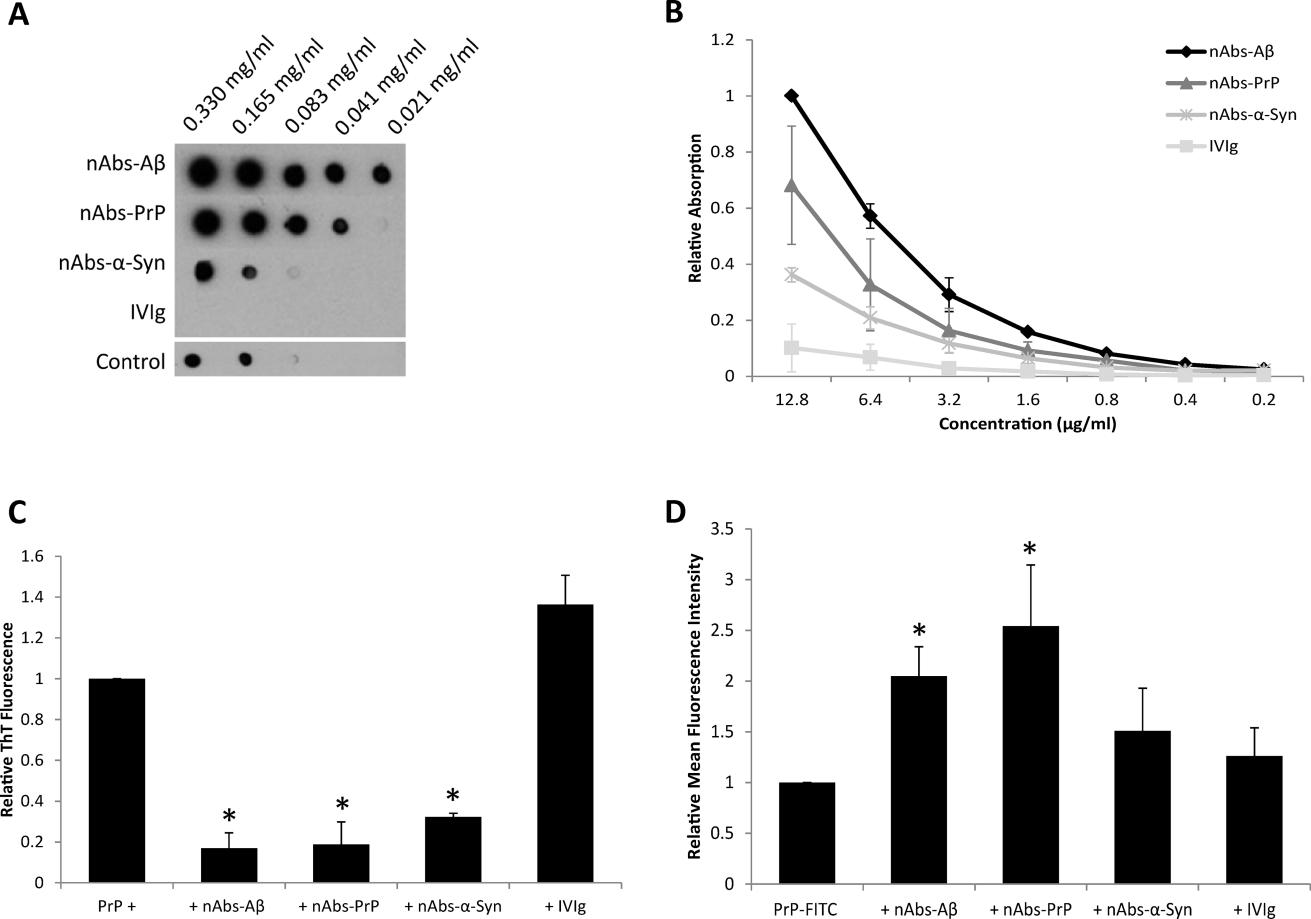
Binding and function of nAbs on PrP. (A) Interaction between nAbs and aggregated PrP fragment in Dot Blot. PrP with AA sequence 106–126 A117V was dotted in a decreasing concentration pattern and incubated with nAbs/ IVIg overnight. As a control, the membrane was incubated with secondary antibody only. Fig A shows one representative out of three independent experiments. (B) Measurement of aggregated PrP fragment binding to nAbs using ELISA. The surface of a 96-well plate was coated with aggregated PrP fragment and incubated with decreasing concentrations of different nAbs/ IVIg. Experiments were normalized to the highest concentration (nAbs-Aβ 12.8 μg/ml). (C) PrP fragment ThT-assay following treatment with different nAbs. PrP fragment was incubated with or without nAbs/ IVIg. Values were normalized to the untreated sample as reference control. (D) Effect of different nAbs on aggregated PrP fragment uptake by primary microglial cells. PrP-FITC fragment was pre-aggregated first and then incubated with or without nAbs/ IVIg. Primary microglial cells were treated with aggregated PrP fragment + nAbs/ IVIg mix. To distinguish between live and dead cells, primary cells were stained with HOECHST 33258. To focus only on microglial cells, cells were stained with a CD11b antibody. C-D show mean values with SD from 3 independent experiments. For every assay IVIg was used as a reference control.

By Dot blot (Fig 2A) and by ELISA experiments (Fig 2B), all nAbs displayed binding to aggregated PrP fragment. nAbs-Aβ and nAbs-PrP showed higher avidity compared to nAbs-α-Syn. IVIg binding was nearly undetectable. Nevertheless, the control staining in Dot Blot showed an unspecific binding signal by using the secondary antibody alone. However, the intensity of this binding was nearly the same to nAbs-α-Syn, which make the interpretation of the Dot Blot assay alone difficult. In further ELISA experiments, a significant effect was observed in nAbs-Aβ (*p* = 0.0001), nAbs-PrP (*p* = 0.00006) and nAbs-α-Syn (*p* = 0.01) experiments compared to IVIg. Surprisingly, SPR analysis revealed no detectable binding of aggregated PrP fragment and nAbs, indicating that the nAbs-corresponding PrP epitope might be sensitive to the immobilization process. Interaction studies with monoclonal 3F4 antibody show a clear binding signal, indicating that the PrP fragment was coated successfully and at least the 3F4 epitope was available (see supporting information S1 Fig).

Using the functional ThT assay, significant inhibition of PrP fragment fibrillation was measured in the presence of all nAbs in comparison to IVIg (Fig 2C, and for time course see supporting information S2 Fig). The peptide fibrillation of PrP fragment was prevented by the treatment with nAbs-Aβ with a power of 83% (*p* = 0.0006), nAbs-PrP with a power of 82% (*p* = 0.0007) and nAbs-α-Syn with a power of 68% (*p* = 0.0007). In microglial uptake experiments, the functional effect was confirmed in the presence of nAbs-Aβ, nAbs-PrP and nAbs-α-Syn (Fig 2D). Significance was found between nAbs-Aβ (*p* = 0.02) and nAbs-PrP (*p* = 0.03) compared to IVIg. In comparison to no additional treatment nAbs-Aβ were able to enhance microglial uptake by a factor of 1.5 and nAbs-PrP by a factor of 1.7. In all experiments we could demonstrate an effect of all nAbs on aggregated PrP fragment binding as well as on a functional level.

### Differential binding and function of α-Synuclein following exposure to nAbs-Aβ, nAbs-PrP and nAbs-α-Syn

**Fig 3:**
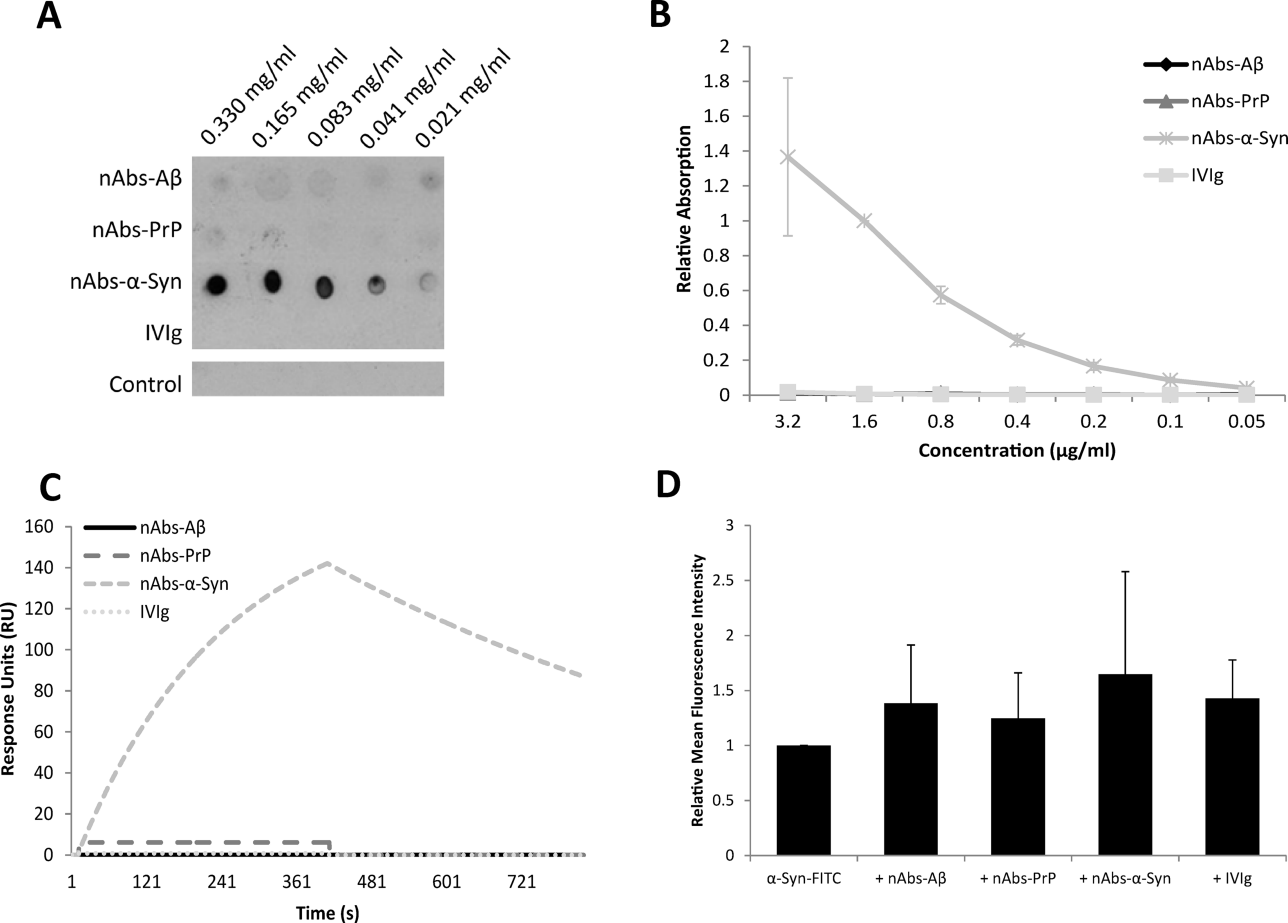
Binding and function of nAbs on α-Syn. (A) Interaction between different nAbs and α-Syn in Dot Blot. α-Syn was dotted in a decreasing concentration pattern and incubated with nAbs/ IVIg. Incubation with the secondary antibody only was used as control. Fig A shows one out of three independent Dot Blots. (B) Interaction of α-Syn with different nAbs using ELISA. The surface of a 96-well plate was coated with α-Syn and nAbs/ IVIg were applied in a decreasing concentration pattern. (C) SPR sensorgram of α-Syn interaction. α-Syn was immobilized as ligand on an SPR chip with about 2000 RUs and nAbs were used as analytes. The association of 0.5 μM nAbs is shown. Interactions are shown after a Langmuir fitting. (D) Microglial uptake of α-Syn using FACS. Primary microglial cells were preincubated with or without nAbs/ IVIg and further additionally incubated with α-Syn-FITC. The data were normalized to that of cells treated with α-Syn-FITC without nAbs. Compared to IVIg, nAbs-treatment could not enhance microglial uptake significantly, but in all cases nAbs and IVIg show a trend to support microglial uptake. B and D Graphs show mean values ± SD of 3 independent experiments. For all experiments IVIg was set as a reference control.

In our experimental setting, only nAbs-α-Syn interacted with α-Syn in Dot Blot assays (Fig 3A) and ELISA experiments (nAbs-α-Syn: *p* = 2.97*10^−11^)(Fig 3B). Neither nAbs-Aβ (*p* = 0.56) nor nAbs-PrP (*p* = 0.47) showed any binding towards α-Syn. Further, the control staining of the secondary antibody alone did not show an unspecific binding. This unique binding to α-Syn by nAbs-α-Syn was confirmed by SPR analysis (Table 1B). nAbs-Aβ and nAbs-PrP did not display any interaction with the α-Syn protein. The corresponding sensorgram illustrated the interaction (Fig 3C). The nAbs-α-Syn interaction curve displays a minimal decreasing dissociation signal, indicating a stable complex between the binding partners.

The results of microglial uptake (Fig 3D) demonstrated that all nAbs as well as IVIg enhance microglial uptake of α-Syn, however, there was no significant difference between nAbs-Aβ (*p* = 0.93), nAbs-PrP (*p* = 0.70), nAbs-α-Syn (*p* = 0.76) and IVIg.

## Discussion

In this study, we investigated the differential affinity of nAbs to the respective aggregating proteins and addressed the question of a putative common epitope of Aβ, PrP and α-Syn for nAbs.

Dot Blot and ELISA experiments demonstrated binding of Aβ and PrP fragment by nAbs-Aβ, nAbs-PrP and nAbs-α-Syn. These results were confirmed by SPR spectroscopy of Aβ and α-Syn. Surprisingly, no PrP interactions were found using SPR-technology. Probably, the structural epitope of PrP, which is recognized by nAbs was hidden by random attachment of the peptide on the SPR chip, or acidic conditions during amine coupling may cause a loss of PrP peptide epitope integrity and therefore prevent interaction with the antibodies`paratope. Other causes could be excluded due to successful testing of monoclonal 3F4 antibody (S1 Fig) and testing of corresponding Aβ and α-Syn.

On the functional level, all three nAbs were found to affect PrP fragment aggregation and uptake as expected from the binding studies. In contrast, functional assays with Aβ did not display the same nAbs efficiency as seen in the binding assays. For microglial Aβ uptake we used the murine microglial cell line BV-2 instead of primary microglial cells. The BV-2 cell line is often used as an alternative system for pMG because of many comparable parameters, such as LPS activation and cytokine secretion [32,33]. In contrast to aggregated PrP fragment the uptake of FITC-conjugated Aβ could not be observed in FACS studies with primary microglial cells. Surprisingly, we also observed small effects on α-Syn uptake with all three nAbs, although we excluded antigen-antibody formation, and we suppose that the general uptake effect of IVIg and other mechanisms apart from Fcγ-receptor mediated microglial uptake might account for that effect [34]. Performing cell culture assays has the disadvantage that underlying cellular mechanisms and proteins involved remain elusive. Due to our focus on antibody related Fcγ-mediated uptake we have not investigated other mechanisms, which could enhance the microglial peptide uptake. To ensure that the observed enhancement is not dependent on the presence of a complete antibody the microglial uptake could be tested only in the presence of F(ab’)2-fragment of the different nAbs. As negative control the Fc part of IVIg (received after a pepsin digestion) could be used to enlighten possible Fc-effector functions of the investigated nAbs. Therefore, we set this assay as a limitation of our study.

Using the here presented assays we were able to determine that the epitope of α-Synuclein exhibits a unique binding affinity for nAbs. The protein was solely detected by nAbs-α-Syn. This phenomenon might be explained by its physiological appearance and its role in the organism. Although Aβ and PrP are extracellular proteins, α-Syn is found intracellularly [35–37]. Furthermore, α-Syn can bind to circulating erythrocytes [38]. If these red blood cells burst by different stimuli a large amount of α-Syn would be available in the blood. A unique formation of specific nAbs-α-Syn could prevent strong immune responses against extracellular α-Syn in the human organism.

In summary, based on the results of antigen-antibody binding, effects on fibrillation *in vitro* and uptake by microglial cells, we provide the idea of a similar structural epitope for Aβ and PrP. α-Syn however, seems to form a different structural epitope, as it is exclusively recognized by nAbs-α-Syn. Nevertheless, all three nAbs show positive functional effects on α-Syn by microglial uptake. Interestingly, nAbs-α-Syn recognize Aβ and PrP fragment. According to Pasquali et al. there might be a structural conformity within the CDR3 region of nAbs-Aβ and nAbs-PrP [39]. Although we could detect strong binding between Aβ and all three nAbs, the effects of nAbs on the functional level were rather small.

In conclusion, nAbs-Aβ und nAbs-PrP recognize similar epitopes, whereas nAbs-α-Syn are able to recognize also the presumed different α-Syn epitope and might be more specific for its antigen. Thus, different proteins with no conformity in their linear AA-sequences might build similar structural epitopes, and different nAbs might be able to bind to this structure rather than to a linear structure. The generation of nAbs against structural instead of linear epitopes shows the “cleverness of the immune system”. In this case, several proteins form nearly similar pathological structures and by this the same antibody epitope, which can be recognized by the same antibodies. For this, we extend the functional characteristics of the investigated nAbs to polyreactive autoantibodies recognizing structures presented by Aβ and PrP instead of monoreactive antibodies. The previous designation such as nAbs-Aβ could be misinterpreted as only Aβ binding antibodies. The investigated nAbs do not only bind the peptide from the purification process; they are polyreactive nAbs, which also detect other proteins with the same structural epitope.

In addition, our results could play an important role in further research on neurodegenerative diseases. In clinical trials, active immunization has failed because of the incidence of encephalitis [40], and passive immunization with monoclonal antibodies against Aβ showed no significant effect [41,42]. However, first passive immunization strategies based on antibodies secreted by human pre-existing B cells deliver promising outcome [43]. The outline of structure-dependent antibodies, as nAbs, provides a new approach for effective antibody development for the treatment of neurodegenerative diseases.

## Supporting information

S1 Fig Positive controls on the different peptides. (A-C) SPR interaction sensorgrams were shown. Peptides were bound covalently onto the chip surface and monoclonal antibodies were set with 50 nM with a decreasing concentration pattern. (A) shows Aβ channel with 6E10 antibody, (B) aggregated PrP fragment with 3F4 antibody and (C) α-Syn with 211 antibody. (D) Dot Blot from different peptides with their related monoclonal antibody as positive control and A11 as confirmation of oligomerization. Peptides were dotted in a decreasing concentration pattern and were incubated with the antibodies after blocking. Bindings were visualized via HRP-conjugated secondary antibody and ECL kit on an X-ray film.
(TIF)

S2 Fig PrP fragment aggregation over 24 hours by ThT assay. PrP fragment was incubated with or without nAbs/ IVIg. 10 μl of the incubation mixtures were taken at time point 0,2,4, and 24 hours to measure the aggregation states. The aggregation was measured at 450 nM using a Tecan Infinite M200.
(TIF)
