# Supplementary figures and images for: Extending the functional characteristics of naturally occurring autoantibodies against β-Amyloid, Prion Protein and α-Synuclein
Source: PLoS One. 2018 Aug 29;13(8):e0202954. doi: 10.1371/journal.pone.0202954 (PMC6114858; doi:10.1371/journal.pone.0202954)

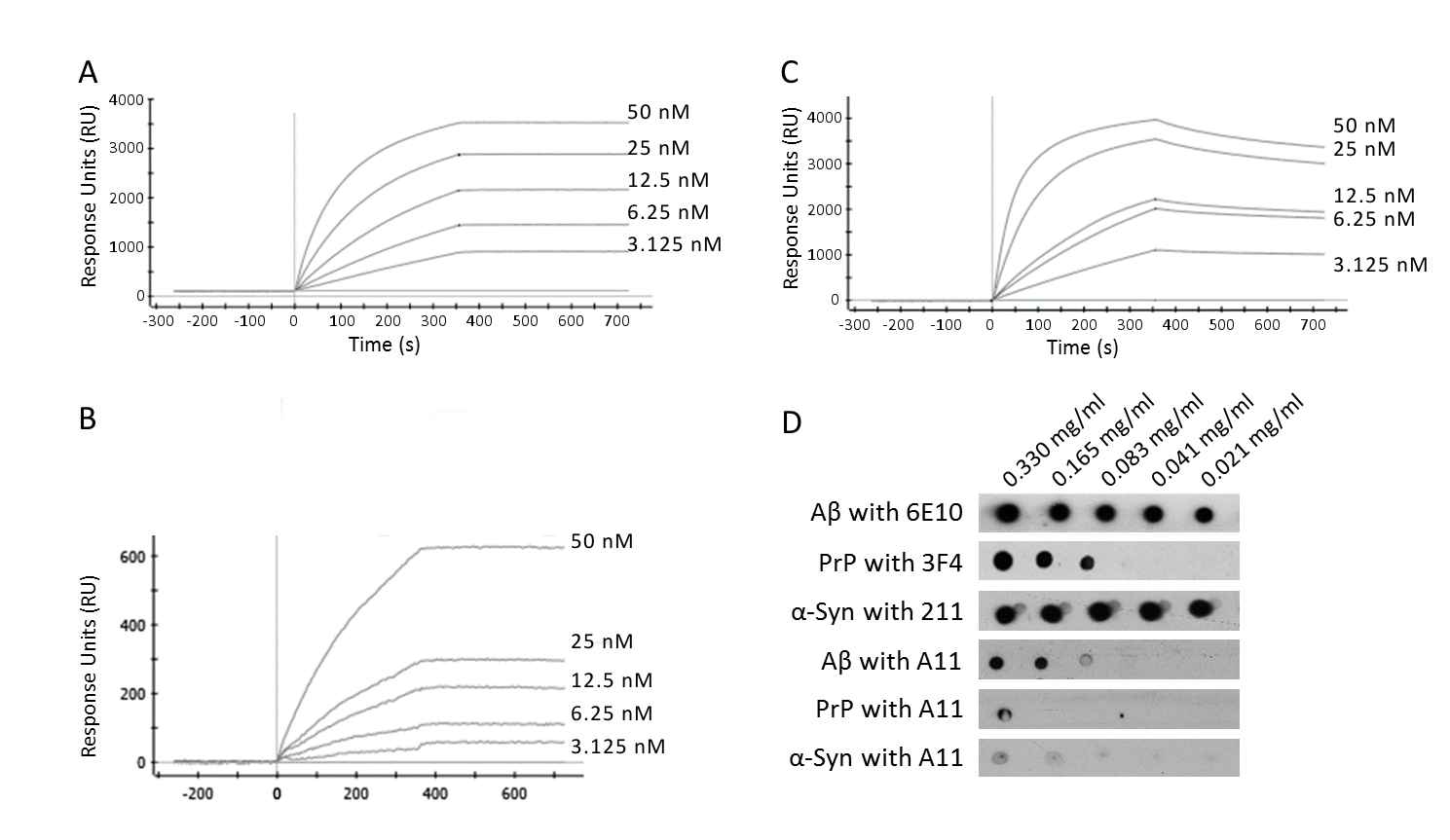

Supplement: S1 Fig — (A-C) SPR interaction sensorgrams were shown. Peptides were bound covalently onto the chip surface and monoclonal antibodies were set with 50 nM with a decreasing concentration pattern. (A) shows Aβ channel with 6E10 antibody, (B) aggregated PrP fragment with 3F4 antibody and (C) α-Syn with 211 antibody. (D) Dot Blot from different peptides with their related monoclonal antibody as positive control and A11 as confirmation of oligomerization. Peptides were dotted in a decreasing concentration pattern and were incubated with the antibodies after blocking. Bindings were visualized via HRP-conjugated secondary antibody and ECL kit on an X-ray film. (TIF) [file pone.0202954.s001.tif]

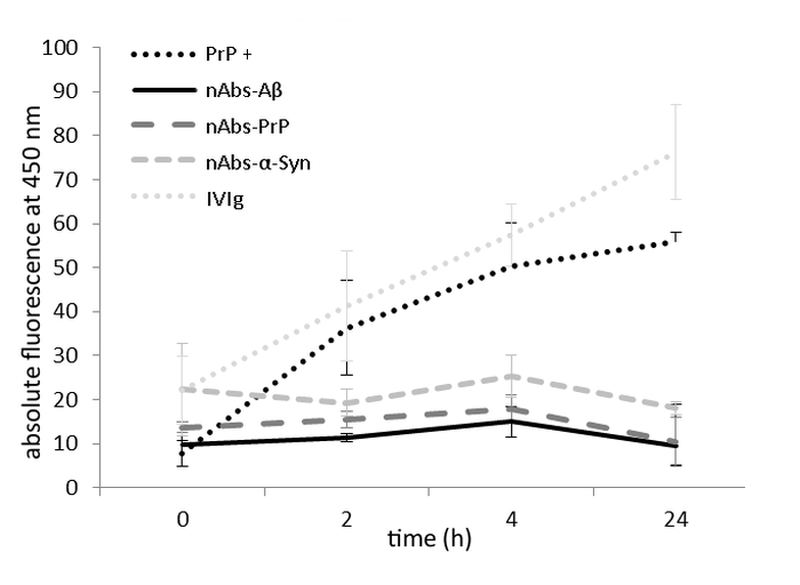

Supplement: S2 Fig — PrP fragment was incubated with or without nAbs/ IVIg. 10 μl of the incubation mixtures were taken at time point 0,2,4, and 24 hours to measure the aggregation states. The aggregation was measured at 450 nM using a Tecan Infinite M200. (TIF) [file pone.0202954.s002.tif]
